# NMR/MRI Techniques to Characterize Alginate-Based Gel Rafts for the Treatment of Gastroesophageal Reflux Disease

**DOI:** 10.3390/gels11090749

**Published:** 2025-09-17

**Authors:** Ewelina Baran, Piotr Kulinowski, Marek Król, Przemysław Dorożyński

**Affiliations:** 1 Institute of Technology, University of the National Education Commission, Krakow, Podchorążych 2, 30-084 Kraków, Poland; ewelina.baran@uken.krakow.pl; 2 Department of Drug Technology and Pharmaceutical Biotechnology, Medical University of Warsaw, Banacha 1, 02-097 Warszawa, Poland; marek.krol@wum.edu.pl; 3 Department of Inorganic Chemistry and Pharmaceutical Analytics, Jagiellonian University Medical College, Medyczna 9, 30-688 Kraków, Poland; przemyslaw.dorozynski@uj.edu.pl

**Keywords:** gastroesophageal reflux disease (GERD), alginate-based raft formulations, magnetic resonance imaging (MRI), nuclear magnetic resonance (NMR) relaxometry, acid reflux, raft formation, antacid formulations, sodium alginate

## Abstract

Gastroesophageal reflux disease (GERD) is associated with symptoms such as heartburn, resulting from gastric content reflux. Alginate-based raft-forming gel formulations represent a non-pharmacological strategy for GERD management by forming a floating gel barrier in the stomach. This study evaluated three commercial anti-reflux oral gel systems under simulated fed-state gastric conditions, using in vitro magnetic resonance relaxometry techniques. Magnetic resonance imaging (MRI) was performed in 0.01 M hydrochloric acid (HCl) to visualize gel raft formation, spatial structure, and spatial distribution of effective T_2_ relaxation time. Nuclear magnetic resonance (NMR) relaxometry in 0.01 M deuterium chloride (DCl) measured T_1_ and T_2_ relaxation times of the protons that were initially included in the preparation to assess its molecular mobility within the gel matrix. Two formulations formed floating, coherent gels, whereas the remaining one exhibited only polymer swelling without flotation. In one case, relaxometry data revealed a solid-like component that can be detected, indicating enhanced mechanical stability. The performance of each formulation was influenced by interactions among alginate, bicarbonates, and calcium ions, which determined gel consistency and flotation behavior. MRI and NMR relaxometry in vitro provide valuable non-invasive insights into the structural and functional behavior of alginate-based gel formulations. This approach supports the rational design of advanced gel-based therapies for GERD by linking molecular composition with in situ performance.

## 1. Introduction

Post-prandial reflux, common in gastroesophageal reflux disease (GERD), often occurs after meals. In healthy individuals, these episodes are brief and asymptomatic, but GERD patients experience them more frequently and with associated symptoms. According to the Montreal consensus, GERD is defined by troublesome symptoms or complications, though not all can be directly attributed to reflux [1]. The hallmark symptom is heartburn, which typically results from reflux of gastric acid into the esophagus, most commonly after meals or during nighttime, and can often be alleviated by lifestyle modifications such as elevating the head of the bed. Regurgitation is another typical symptom, and although both heartburn and regurgitation are specific for GERD, they are relatively insensitive markers of reflux. GERD symptoms frequently worsen in the recumbent position, particularly postprandially. Dysphagia may also be present in uncomplicated GERD; however, its occurrence warrants a thorough evaluation, as it can indicate strictures, rings, malignancy, or esophageal motility disorders. Chest pain is sometimes attributed to GERD but should prompt exclusion of cardiac causes before a reflux etiology is assumed. Additional symptoms include dyspepsia, nausea, bloating, sore throat, globus sensation, and epigastric pain [2,3,4].

Globally, GERD prevalence varies significantly across regions. In North America, estimates range from 18.1% to 27.8%, while in Europe they fall between 8.8% and 25.9%. East Asia has a lower prevalence of 2.5% to 7.8%, whereas the Middle East shows greater variability, with estimates ranging from 8.7% to 33.1%. In Australasia, prevalence is reported at 11.6%, while in South America it is around 23%. Recent Global Burden of Disease (GBD 2019) data further highlight the growing impact of GERD: in 2019, Paraguay reported the highest age-standardized disability-adjusted life years (ASDR) at 125.9 per 100,000, while China reported the lowest at 34.9 per 100,000. Between 1990 and 2019, GERD burden increased globally, driven primarily by population growth (≈70% of the increase) and aging (≈24%). Projections indicate that from 2019 to 2030, the total global disability-adjusted life years attributable to GERD will rise by 21.3%, with the steepest increase expected in low-middle sociodemographic index (SDI) regions (≈61%), underscoring GERD as a persistent and growing global health challenge [5,6,7].

The strong association between acid reflux and heartburn has led to widespread self-treatment strategies using antacids, anti-reflux agents, and over-the-counter H2-receptor antagonists. Antacids, such as sodium bicarbonate and magnesium hydroxide, offer rapid relief by neutralizing stomach acid within minutes. However, their effects are short-lived, often requiring repeated doses due to ongoing acid production. In contrast, H2-receptor antagonists (e.g., cimetidine and ranitidine) take longer to act but provide more sustained acid control, lasting around six hours [8,9,10].

A simple, non-pharmacological alternative for managing GERD is the use of alginate gel rafts, which offer mechanical protection against reflux [2,11,12,13]. Alginate is a polymer composed of D-mannuronic acid (M) and L-guluronic acid (G), linked by 1,4-glycosidic bonds. Alginates form hydrogels that swell as solvent molecules penetrate the polymer, increasing its volume without dissolving the macromolecules [14]. In the acidic environment of the stomach, alginate salts and alginic acid rapidly form a low-density, viscous gel, which, in vitro, forms within seconds and within a few minutes in vivo. Alginate formulations often include bicarbonate, which produces CO_2_ bubbles that create a floating foam or “raft” on the gastric contents, aiding in acid neutralization [2,15].

Gel raft formulations for oral administration (GRFFOA) are not widely used, and as a result, methods for evaluating their performance are still being refined. Keppler et al. assessed alginate-based raft-forming formulations’ strength, resilience, and pH profile under simulated gastric conditions [14]. Gel rafts were formed in either hydrochloric acid (HCl) solution or food systems, matured at 37 °C, and tested for strength using a texture analyzer to measure peak force during compression. Raft resilience was assessed by incubating rafts in the media of varying pH levels, and their mass was measured over time to determine disintegration.

While some products demonstrate rapid pH elevation, sustaining neutralization can be challenging, particularly when the raft structure is disrupted. Tests suggest that while neutralization within the raft may persist, the bulk pH remains largely unaffected. Additionally, antacids such as aluminum hydroxide may improve acid-neutralizing capacity but reduce raft strength. For pH profiling, rafts were stirred with added HCl to simulate stomach movements, with pH measured until reaching non-acidic reflux at pH 4 [16].

Raft strength, measured by resistance to movement through the gel, varies between formulations. Despite the inclusion of antacids like calcium carbonate or magnesium carbonate, the clinical performance of alginate-based systems often exceeds what could be explained by their acid-neutralizing properties alone. Tests reveal that variables such as pH gradients within the raft and resistance to simulated reflux can differ significantly across products.

More advanced techniques have been employed to characterize alginate-aloe vera raft-forming systems. Mechanical properties are analyzed through oscillatory strain sweep tests to assess viscoelastic behavior, while differential scanning calorimetry (DSC) and Fourier-transform infrared spectroscopy (FTIR) offer insights into their thermal properties and chemical structures [16]. Despite comprehensive evaluations of their physical, chemical, and mechanical properties, the exact mechanisms behind the clinical efficacy of alginate-based raft-forming systems, particularly in their interaction with gastric contents and symptom relief, remain unclear [14]. While these tests are useful for evaluating functionality, they do not explain the molecular mechanisms responsible for the swelling and flotation of rafts.

An important methodological aspect in studies of raft-forming gel preparations is the pH of the medium used to simulate the gastric environment. Typically, highly acidic media—such as 0.1 mol/L hydrochloric acid—are employed to model fasting gastric conditions [14,17,18,19]. However, patient information leaflets for commercial formulations often recommend administering these products after meals, a state associated with a more diluted gastric acid environment, typically around 0.01 mol/L HCl.

MRI and NMR, often combined with techniques like micro-computed tomography (micro-CT), have been successfully employed to analyze the hydration of hydrophilic polymer matrix systems, commonly in tablet form [20,21,22]. These studies show that various macroscopic and molecular phenomena govern the functional properties of these dosage forms [23]. Magnetic resonance imaging (MRI) techniques primarily monitor water distribution and mobility within matrix systems [20,24,25]. Most MRI studies on hydrophilic polymer hydration utilize spin-echo sequences, like relaxometry and diffusometry, often performed in water [25]. MRI has been used to study alginate gels and solutions [26,27]. Another approach using low-field NMR relaxometry (LF NMR) has expanded the ability to observe changes in polymeric systems. Recently, Baran et al. applied LF NMR to track mass transport in alginate tablets [28]. By hydrating tablets with both water (H_2_O) and heavy water (D_2_O), it becomes possible to observe solvent penetration and the evolution of both the polymer and the water that is initially incorporated into the matrix [28]. LF NMR relaxometry provides volumetric information and measures T_1_, T_2_, or both relaxation times. In heterogeneous samples, different regions exhibit varying relaxation times, producing T_1_-T_2_ relaxation maps that separate proton pools by signal components. NMR relaxometry offers detailed insights into the composition and structure of the sample and can be used to study sodium alginate gelation [29]. Combination of MRI and LF NMR can further expand research capabilities [30]. MRI has also been used for in vivo raft studies [31,32]. In vivo studies are very important regarding the assessment of the real effectiveness of the therapeutic agent. However, in vivo MRI studies are prone to motional artifacts, and the results also depend on the individual. To compare preparations, in vitro techniques also seem to be optimal because of a much wider spectrum of techniques available, including spatially resolved (MRI) and non-spatially resolved (LF NMR relaxometry) [30]. Considering the above, it can be expected that these methods are well-suited for studying mechanisms of gel systems evolution: in this particular case, formation and assessment of gel raft properties, as they allow non-invasive in situ measurements.

Considering the above, it can be expected that advanced imaging techniques, such as relaxometric magnetic resonance imaging (MRI) and nuclear magnetic resonance (NMR) relaxometry, are well-suited methods for studying the mechanisms of gel system evolution (formation and assessment of gel raft properties), as they allow for non-invasive in situ measurements.

The goal of this work was to deepen the understanding of GRFFOA by utilizing magnetic resonance relaxometry in vitro, with both spatially localized (MRI) and non-spatially localized (LF NMR) techniques, to evaluate and characterize the physicochemical properties of commercial formulations applied in GERD treatment. Through MRI and NMR relaxometry, we aimed to investigate the behavior of these formulations under simulated gastric conditions according to commercial product leaflets and to explore the mechanisms underlying their effectiveness in reducing reflux and its associated symptoms.

## 2. Results and Discussion

The products selected for testing represent some of the most popular alginate/antacid formulations available on the market. Table 1 provides detailed information about the compositions of the tested GRFFOA. The choice to study real commercial formulations instead of model ones is justified by the need to capture the actual complexity, variability, and performance characteristics of products as they are used clinically. Real formulations, such as Alugastrin MAX Protect (formulation A), Gaviscon (formulation B), and Gastrotuss (formulation C), contain not only primary functional components like alginates and bicarbonates but also diverse excipients—e.g., surfactants, preservatives, viscosity modifiers, and flavorings—which can significantly influence raft formation, mechanical stability, and flotation behavior. By using authentic products, the study provides insights that are directly relevant to patient outcomes and therapeutic efficacy, especially under realistic gastric conditions (e.g., postprandial pH of 0.01 M HCl), as recommended in commercial product leaflets. This approach ensures that conclusions about structural behavior, relaxation properties, and functional performance reflect the actual behavior of medications in vivo, rather than idealized or simplified models that may overlook critical formulation-dependent effects.

### 2.1. Initial Characteristics of Raft Formulations

In all the tested formulations, the main component responsible for raft formation was an alginic acid salt—sodium alginate in Formulations A and B, and magnesium alginate in Formulation C. The role of alginates was reinforced by viscosity-control polymers, namely polyacrylic acid (Formulation A), Carbomer 974P (Formulation B), and xanthan gum (Formulation C), which acted as thickeners and stabilizers of the gel structure. Effervescence and buoyancy were achieved through gas-forming agents, with sodium and calcium bicarbonates applied in Formulations A and B and sodium bicarbonate alone in Formulation C. The presence of calcium ions further stabilized the raft hydrogel by cross-linking alginate chains into the characteristic “egg-box” structure [13]. In Formulation C, the core raft system of magnesium alginate, xanthan gum, and sodium bicarbonate was complemented by simethicone, which acted as a surfactant and antifoaming agent, reducing bubble coalescence and ensuring raft uniformity. To ensure microbiological stability, all formulations contained methyl- and propylparaben, while flavoring and sweetening agents differed: sodium saccharin with synthetic or mint flavors in Formulations A and B, and natural flavors, honey, and fructose in Formulation C; the latter also contributed demulcent properties. Sodium hydroxide was included in Formulations B and C as a pH adjuster, enabling Carbomer chain relaxation and gel formation in the former. Formulation C was enriched with D-panthenol, zinc oxide, and extracts of *Althaea officinalis* and *Papaver rhoeas*, which provided mucosal protection, soothing effects, and healing support. Purified water served as the vehicle across all formulations.

**Figure 1:**
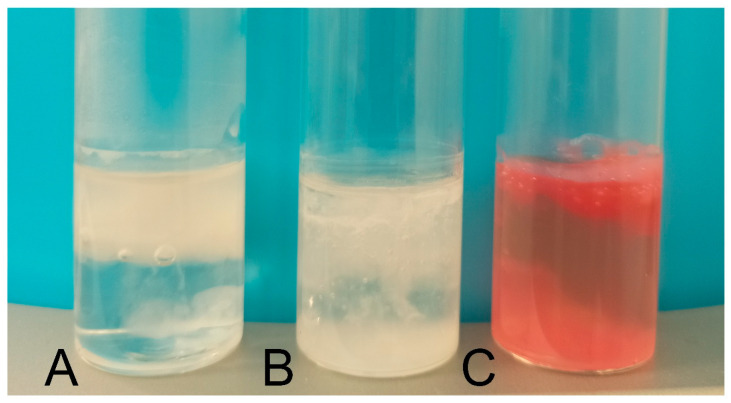
The samples of gel raft formulations in the fed state simulated gastric fluid, i.e., in 0.01 M deuterium chloride (DCl): (**A**) Formulation A; (**B**) Formulation B; and (**C**) Formulation C.

The visual observations of gel raft formation after application in fed-state simulated gastric fluid revealed noticeable differences between the formulations. Formulation A and Formulation B formed a floating gel layer on the surface of the solution, while Formulation C sank almost completely. In the cases of Formulation A and Formulation B, the carbonates in these products reacted with the acidic environment, releasing CO_2_, which became incorporated into the polymeric material. For Formulation B, the release of gas caused partial disintegration of the raft, with some material settling at the bottom. Figure 1 shows samples of gel raft formulations in a hydrochloric acid solution (0.01 M), simulating fed-state gastric fluid. In Formulation C, the presence of simethicone influenced bubble formation and the floating characteristics of the raft. Additional details regarding the visual observations of raft behavior are provided in Table 2.

Macroscopically, gel rafts should be adequately cohesive, possess flotation properties, and have the ability to swell. They should also exhibit sufficient mechanical resistance to maintain their functional characteristics despite the peristaltic forces in the stomach. In every case, the material ingested after a meal forms a hydrogel layer on the surface of the gastric contents, creating an anti-reflux barrier and protecting the esophagus from the regurgitation of stomach contents. The MRI revealed certain differences in the behavior of the preparations when placed in a 0.01 M hydrochloric acid solution simulating the properties of gastric juice after a meal.

### 2.2. Magnetic Resonance Imaging

**Figure 2:**
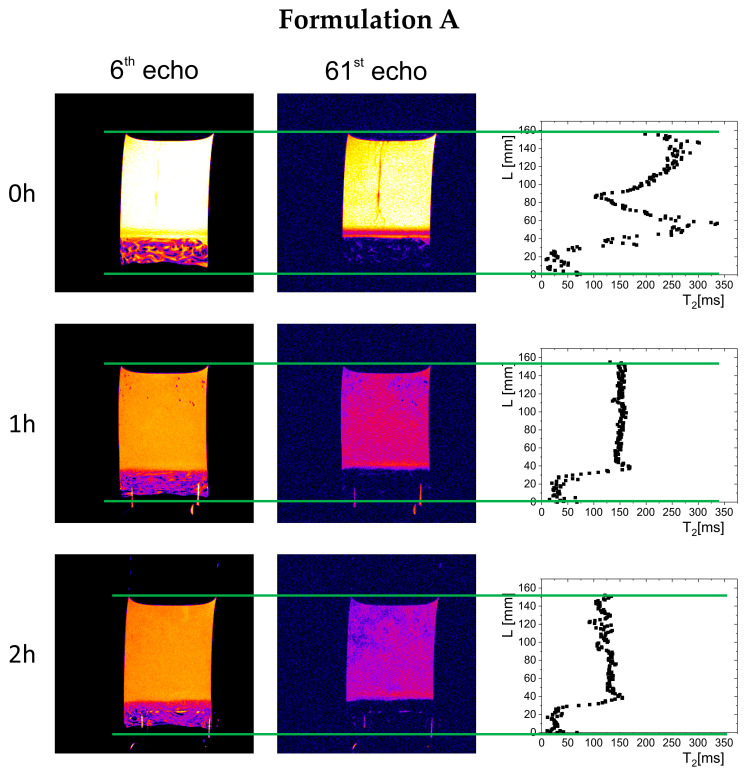
MRI images of the Formulation A specimen in simulated gastric juice after a meal (0.01 M HCl solution). Left column: images of the specimen at 6th echo (presenting the material with contribution from signal components characterized by both short and long T_2_ relaxation times), central column: images of the specimen for 61st echo (presenting gel raft image with contribution from signal components characterized by relatively long T_2_), right column: T_2_ profile on the sample cross-section. Green lines indicate the bottom and top of the sample.

**Figure 3:**
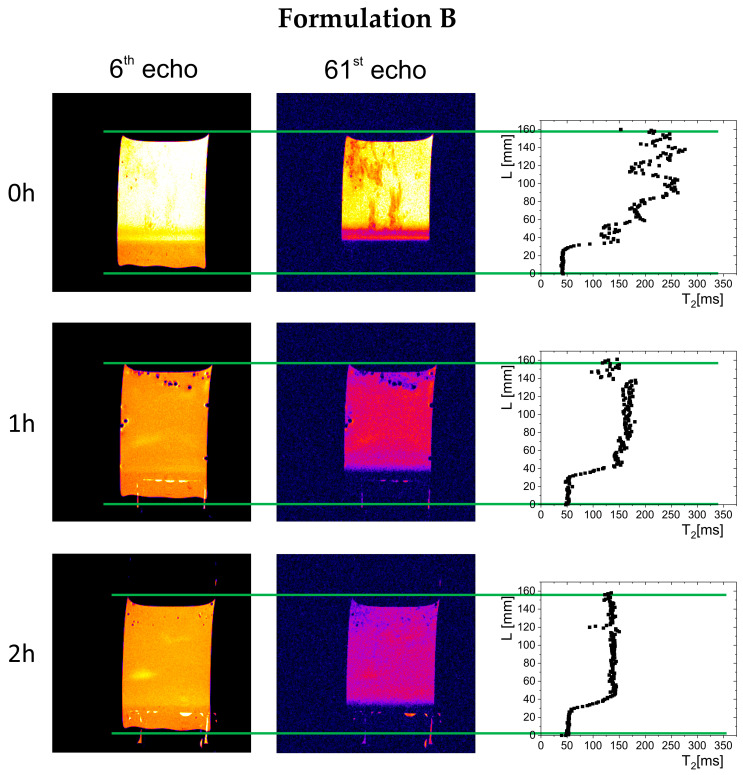
MRI images of the Formulation B specimen in simulated gastric juice after a meal (0.01 M HCl solution). Left column: images of the specimen at 6th echo (presenting the material with contribution from signal components characterized by both short and long T_2_ relaxation times), central column: images of the specimen for 61st echo (presenting the raft image with contribution from signal components characterized by relatively long T_2_), right column: T_2_ profile on the sample cross-section. Green lines indicate the bottom and top of the sample.

**Figure 4:**
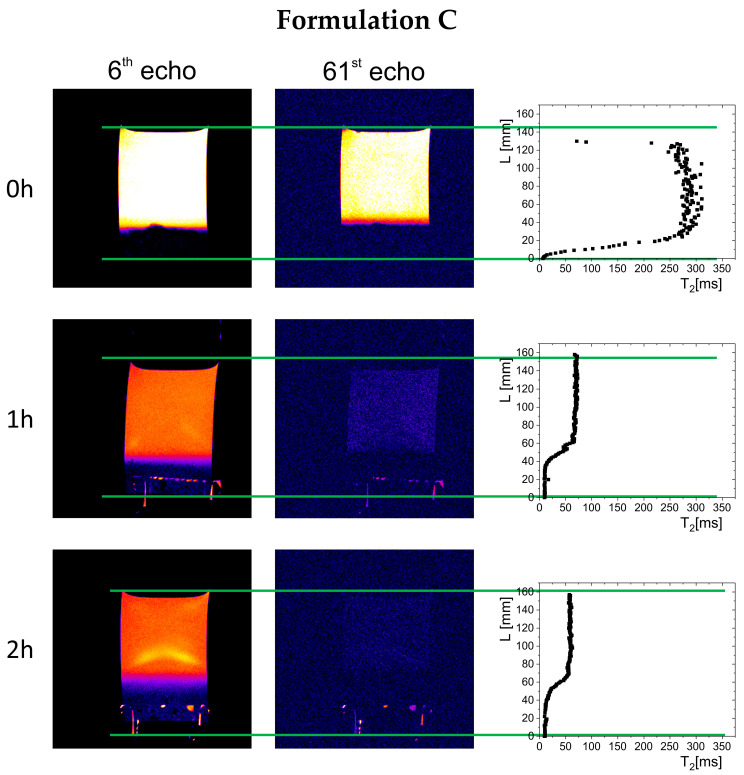
MRI images of the Formulation C specimen in simulated gastric juice after a meal (0.01 M HCl solution). Left column: images of the specimen at 6th echo (presenting the material with contribution from signal components characterized by both short and long T_2_ relaxation times), central column: images of the specimen for 61st echo (presenting the raft image with contribution from signal components characterized by relatively long T_2_), right column: T_2_ profile on the sample cross-section. Green lines indicate the bottom and top of the sample.

The results of magnetic resonance imaging in a 0.01 M hydrochloric acid solution are presented in Figure 2, Figure 3 and Figure 4. The images acquired at 6 and 61 echo show exactly the same spatial region but with a different contrast, emphasizing different features of the imaged object. Additionally, in the right column are calculated T_2_ profiles on the sample cross-section. In all cases, immediately after immersion, part of the material sank to the bottom of the vessel, forming a polymer depot with a varied structure (left column in Figure 2, Figure 3 and Figure 4). For Formulation A, a non-homogeneous material structure at the bottom of the vessel was observed, differing from the structures formed in the cases of Formulation B and Formulation C. Immediately after the measurements began (hour 0), gel raft formation was observed in the samples of Formulation A and Formulation B, which manifested by spatially varying T_2_ along the cylindrical vessel axis (right column in Figure 2, Figure 3 and Figure 4). The spatial T_2_ variability was caused by the movement of polymer material, which, due to the release of carbon dioxide from sodium bicarbonate and calcium carbonate, was lifted toward the surface of the simulated gastric fluid. A different picture was observed for Formulation C, where the T_2_ profile showed a region of material deposited at the bottom of the vessel—characterized by relatively low T_2_ values (<50 ms)—and a solution region of higher molecular mobility, and thus longer T_2_ relaxation times (250–300 ms). In subsequent hours, a floating raft was observed for Formulation A and Formulation B, as well as a gradual expansion/dissolution of the polymer material in Formulation C, which manifested as a shortening of the solution’s T_2_ relaxation time (≥60 ms).

Over time, a gradual evolution in the thickness of the material deposited at the bottom of the vessel was observed, related to phenomena occurring inside the preparations—raft flotation, polymer swelling, and changes in physicochemical properties reflected in the spatial distribution of T_2_ relaxation times. In the initial phase, the thickness of the deposited polymer layer was 11.2 mm for Formulation A, 6.8 mm for Formulation B, and 4 mm for Formulation C. After one hour, the thickness of the layer in Formulation A decreased to 7 mm and remained unchanged until the end of the study, indicating that part of the material had been lifted and incorporated into the gel raft formed on the surface. The Formulation B depot increased to 8.3 mm, which may indicate that some material fell from the gel raft, down to the bottom of the vessel. In the case of Formulation C, the polymer layer deposited at the bottom of the vessel gradually increased to 10 mm after one hour and 12 mm after two hours.

Visual and MRI observations of raft formation in fed-state simulated gastric fluid (0.01 M hydrochloric acid solution) revealed differences between formulations. Formulation A and Formulation B formed a floating gel layer on the surface, while Formulation C sank almost completely. In the case of Formulation A and Formulation B, the carbonates in the products reacted with the acidic environment, releasing CO_2_, which became incorporated into the polymeric material. For Formulation B, the gas release led to partial disintegration of the gel raft, causing some material to settle at the bottom.

Despite these differences in flotation, all the preparations successfully formed a hydrogel layer that can act as an anti-reflux barrier. The behavior of the rafts, including their flotation and mechanical integrity, is detailed in Figure 1 and Table 2, which summarize the visual and functional properties of the tested gel systems.

Studies on raft systems are typically conducted in 0.1 M HCl, as it simulates gastric conditions for evaluating raft formation, integrity, buoyancy, and acid neutralization. This acidic medium facilitates calcium ion cross-linking with sodium alginate, forming a stable gel structure, while sodium bicarbonate reactions generate CO_2_ bubbles, ensuring flotation. Testing in 0.1 M HCl also assesses raft stability, prolonged drug release, and effective acid buffering, optimizing in vivo performance [18,19]. However, studies have also explored the effects of different acid concentrations. For instance, one study assessed raft strength using 0.07 M and 0.05 M HCl alongside 0.1 M HCl to observe variations in raft characteristics [17].

In our study, we used 0.01 M HCl, which more accurately represents the fasted-state gastric acid concentration, a standard for raft application. This allows for a more realistic evaluation of gel systems behavior under fasting conditions, where lower acid concentrations may influence raft formation, strength, and duration of action. By simulating the fasted gastric environment, our study provides insights into gel raft performance under the conditions that are most relevant to therapeutic applications.

### 2.3. Low-Field NMR Relaxometry

The MRI performed in HCl solution gave spatially resolved results, but the acquired signal contribution was restricted to the mobile proton pool with long T_2_ relaxation times (echo time of 10 ms). LF NMR measurements were performed in DCl. Using DCl as a medium for non-spatially localized measurement allowed for restricting the detected pool of protons to those originally included in the preparation. Additionally, an echo time of 60 μs also allowed for the acquisition of the signal components with T_2_ s of about 1 ms and shorter, which cannot be detected in MRI experiment.

**Figure 5:**
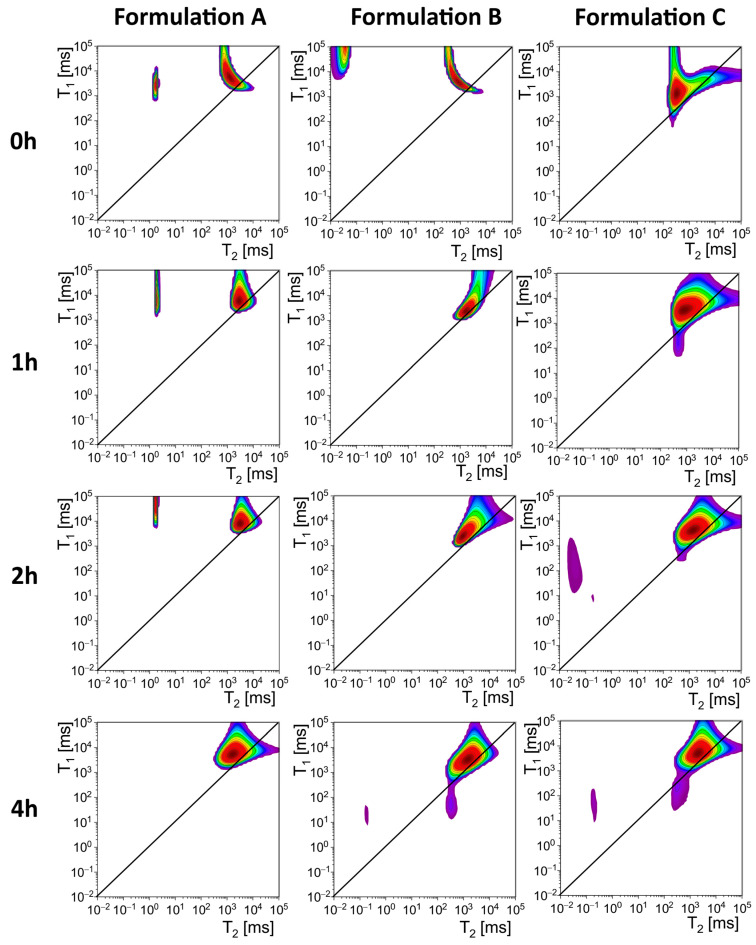
Results of relaxometric tests of Formulation A, Formulation B and Formulation C in DCl solution (0.01 M) immediately after immersion (0 h) and after 1 and 4 h.

The results of NMR relaxometry are presented in Figure 5 and Table 3. The table presents relaxation times (T_1_ and T_2_) and their ratios (T_1_/T_2_) for Formulation A, Formulation B, and Formulation C at specified time intervals (0, 1, 2, and 4 h). Data is reported separately for two peaks, Peak 1 and Peak 2, corresponding to a distinct pool of protons within each sample. These measurements were indicative of the molecular dynamics of the samples.

For Formulation A, Peak 1 showed an initial T_2_ value of 1.88 ms and a T_1_ value of 2799 ms, resulting in a T_1_/T_2_ ratio of 1489 at 0 h. Over time, T_1_ increased substantially, reaching 88,414 ms at 2 h. Peak 1 was not present at 4 h. Peak 2 exhibited an initial T_2_ value of 1274 ms and a T_1_ value of 5301 ms, with a T_1_/T_2_ ratio of 4.16. Over time, these values fluctuated, with T_2_ decreasing initially but increasing at 2 h. By 4 h, T_2_ reached 1650 ms.

For Formulation B, Peak 1 was observed only at 0 h. It was absent at later time points. Peak 2 showed T_2_ and T_1_ values of 867 ms and 3783 ms, respectively, at 0 h, with a T_1_/T_2_ ratio of 4.36. At 1 h, T_2_ increased to 1665 ms, while T_1_ decreased to 2475 ms, reducing the T_1_/T_2_ ratio to 1.49. By 2 h, T_2_ and T_1_ stabilized at 938 ms and 2408 ms, respectively, with a T_1_/T_2_ ratio of 2.57.

For Formulation C, Peak 1 lacked data for the initial time points and only appeared measurable at 2 h, with T_2_ and T_1_ values of 0.04 ms and 174 ms, respectively. By 4 h, T_2_ and T_1_ increased slightly, reaching 0.19 ms and 49 ms. Peak 2 data is available across all time points, with an initial T_2_ value of 318 ms and a T_1_ value of 1319 ms at 0 h, yielding a T_1_/T_2_ ratio of 4.15. Over time, T_2_ and T_1_ increased consistently, reaching 2001 ms and 5019 ms, respectively, by 4 h.

All samples used in the studies were initially in the form of suspension, so from the very beginning of each relaxometry study, a significant pool of protons of high mobility (Peak 2) was visible in all preparations with T_1_/T_2_ close to one, with T_2_ relaxation times ranging from 318 for Formulation C to 1274 ms for Formulation A at 0 h (Figure 5). These long T_2_ times indicated the presence of a highly fluid hydrogel structure. In the case of Formulation A and Formulation B, some of the protons in the material had significantly shortened T_2_ relaxation times, with values below 100 ms and T_1_/T_2_ > 1000 (Peak 1). It suggested that there are protons in the preparation with properties similar to those of an elastic solid-like component. Importantly, in the case of Formulation A and Formulation B, the pool of protons with short T_2_ times appeared after the first hour of contact with the artificial gastric juice solution after a meal, which is likely related to the cross-linking of alginate by calcium ions released from calcium carbonate under the influence of acid. Peak 1 was not observed in the samples’ measurements before they were poured into the DCl solution. This pool of protons, characterized by short T_2_ times, disappeared in the following hours of observation. In Formulation A, peak 1 disappeared after 4 h of observation, whereas for Formulation B it disappeared after 1 h after adding it to the DCl solution. In the case of Formulation A, a pool of protons characterized by short T_2_ relaxation times was also observed. However, this pool remained present throughout the two-hour observation period, unlike Formulation B. In Formulation C, peak 1 occurred only after 2 h of observation. The presence of protons with properties characteristic of a solid-like component makes Formulation A less prone to dispersion and dilution by the food content in the stomach, which translates into the functional properties of the respective formulations. In vivo imaging studies listed by Mandel et al. confirm that alginate-based formulations rapidly form floating rafts that can remain in the stomach for up to four hours [2].

On the other hand, although the studies conducted by Hampson et al. [17] demonstrated that raft resilience and strength are influenced by the alginate dose, as well as calcium and sodium bicarbonate content, the in vivo studies confirmed the functionality of different formulations. The different formulations formed stable rafts that persisted longer than ingested food, effectively suppressing reflux.

### 2.4. Comparison with Previous Studies

The work aimed to perform a relaxometric study of raft-forming commercial preparations in vitro. To the best of our knowledge, in vitro LF NMR/MRI raft studies are absent. The methodology described in the Section 4 is an adaptation and combination of the methods used for studying various aspects of alginate-based compressed matrix hydration [22,28]. In the current study, these methods are used in a different context. The main difference is that raft-forming preparations in their original form contain a dominating mobile pool of protons, unlike previously studied alginate matrix tablets, which contain immobile proton fractions. In the current study, MRI data yielded one-dimensional T_2_ relaxation times obtained across the vessel axis, as depicted in Figure 2, Figure 3 and Figure 4. LF NMR resulted in T_1_-T_2_ correlations (maps) presented in Figure 5.

The only comparison that can be made regarding the methods used in the study is with in vivo studies, as mentioned in the introduction [31,32]. In the study by Sweis et al., raft-forming alginate has been monitored inside the gastric volume after ingestion, using MRI [31]. In the very recent in vivo study by Hoad et al., the authors assessed T_2_ relaxation times in ROIs selected at the upper and lower parts of the raft formed [32]. The authors have noted that this was only possible for two participants. The reason is that in vivo MRI of the gastrointestinal tract (including the stomach) suffers from motional artifacts generated by breathing, stomach peristalsis, and involuntary motions of the volunteer. Nevertheless, such in vivo investigations are highly relevant for assessing the real effectiveness of a therapeutic agent. However, they are also dependent on individual variability.

In contrast, for the purpose of comparing preparations, an in vitro study provides more reliable quantitative results and controlled experimental conditions, allowing for more extensive studies at various pH levels of the medium, as well as at the potential presence of a meal. Additionally, in vitro magnetic resonance techniques appear more suitable, as they allow access to a much broader spectrum of methods, including not only spatially resolved (MRI) but also non-spatially resolved (LF NMR relaxometry) approaches. The methodology used in the current study combines both approaches. Also, using 0.01 M HCl in the MRI study and 0.01 M DCl in LF NMR study allows studying different physicochemical aspects. This methodological approach constitutes a major novelty of the study.

It should be noted that studies on pharmaceutical modified-release matrix tablets have shown that MRI, coupled with innovative biorelevant drug release, is an excellent tool for developing such formulations. Temporal and/or spatiotemporal in situ analysis of macrostructural and physicochemical properties gives hints regarding biorelevant drug-release results [23]. Taking into account the results of the presented study, it can be expected that the development of new GRFFOA can also be supported by NMR/MRI in situ studies in an environment mimicking physiological conditions.

## 3. Conclusions

This study represents the first attempt to quantitatively investigate in vitro raft-forming gel anti-reflux formulations using non-invasive magnetic resonance techniques, including imaging and relaxometry. Also, the measurements were performed in more realistic simulated fed-state conditions regarding pH (0.01 M HCl or DCl) according to product leaflet indications. It demonstrated that raft-forming gel formulations can be effectively studied in situ, revealing changes in physicochemical properties reflected in T_1_ and T_2_ relaxation times. This approach opens up new opportunities for further research, focusing on better simulation of physiological conditions: not only pH, but also the presence of a meal on raft formation. As a consequence, magnetic resonance techniques could play an important role in the development of new raft-forming gel formulations.

Formulation A, Formulation B, and Formulation C display notable differences in their composition and behavior when tested in simulated fed-state gastric fluid. Both Formulation A and Formulation B form gel rafts that float on the surface, providing a protective barrier. These rafts swell upon contact with the liquid, playing a crucial role in reducing acid reflux symptoms. Relaxometry studies reveal molecular distinctions between these products, which may influence their functional characteristics.

One observation from the studies is that Formulation A exhibits the presence of a solid-like component, which could contribute to the durability of its raft. This is likely a result of the interaction between its ingredients, such as alginate and calcium bicarbonate, leading to the formation of a cross-linked raft structure. This may result in a stable and longer-lasting raft that helps maintain its integrity for an extended period, offering consistent protection.

In general, Formulation A and Formulation B gel raft formation involves the release of carbon dioxide as bicarbonates react with gastric acid, allowing the rafts to float. However, the mechanical strength and persistence of the gel rafts may vary between formulations, which can be indirectly reflected in relaxometry results (T_1_ and T_2_ relaxation times). For instance, the existence of a component that can be attributed to solid-like components in the raft formed by Formulation A could suggest stronger mechanical properties, while the rafts of other preparations, such as Formulation B and Formulation C, may dissolve at different rates over time.

These molecular and structural differences suggest that each product offers its own unique strengths depending on individual patient needs. The ability to maintain a stable raft for a longer duration may contribute to the effectiveness of these treatments, offering support for individuals managing gastroesophageal reflux symptoms. The overall performance of each preparation depends on various factors, including how they interact with the gastric environment and their capacity to provide sustained protection.

## 4. Materials and Methods

For this study, three oral alginate-based anti-reflux gel formulations available on the Polish market were selected: Formulation A—Alugastrin Max Protect (Biofarma, Mereto di Tomba, Italy); Formulation B—Gaviscon (Reckitt, Slough, UK); Formulation C—Gastrotuss (D.M.G. Italia S.r.l., Pomezia, Italy). Heavy water (D_2_O) and heavy hydrochloric acid (DCl) were supplied by Sigma-Aldrich (St. Louis, MO, USA). All other materials were of analytical grade purity.

### 4.1. Magnetic Resonance Imaging

For magnetic resonance imaging, 2 mL raft samples were placed in a glass bottle filled with 10 mL of HCl (0.01 M). The bottle was placed in a thermostatic water bath (LW 502 M, AJL Electronic, Kraków, Poland) at a temperature of (37 °C ± 1 °C). Then, it was periodically removed from the water bath for MRI scanning. Images were taken immediately after immersion (0 h) and after 1 and 2 h of incubation. The studies were performed using a Bruker Biospec experimental scanner with a 9.4 T magnetic field (Bruker, Ettlingen, Germany). A multi-spin echo sequence (system sequence name: MSME) was used with the following parameters: echo time (TE) = 10 ms, number of echoes (NE) = 128, repetition time (TR) = 4 s, acquisition buffer size (SI) = 256, number of phase steps = 256, number of signal accumulations (NA) = 1, slice thickness = 1 mm, field of view (FOV) = 50 mm × 50 mm. After reconstruction, separate images of 256 × 256 pixels were obtained for each echo time (n*TE). The image resolution was 0.19 mm.

To obtain 1D images (spatial profiles) of effective T_2_ relaxation times, a procedure similar to that described in detail in the work by Baran et al. (including diagram) [30]. MR images acquired at consecutive echo times were used to generate one-dimensional T_2_ relaxation time profiles across the vessel axis. Each individual fit was based on 128 data points at a given cross-section point. Pixel image intensity vs. echo time for each spatial position was fitted using the Levenberg−Marquardt algorithm with a monoexponential function, and only fits with an R^2^ coefficient greater than 0.95 were included in the final result. The procedure was performed using OriginPro 2024 software package (OriginLab Corporation, Northampton, MA, USA).

### 4.2. Low-Field NMR Relaxometry

Low-field NMR relaxometry was conducted using a 23 MHz NMR Rock Core Analyzer with PROSPA 4.26 software package (Magritek, Aachen, Germany). For measurement purposes, samples were placed in glass tubes containing 5 cm^3^ of deuterium chloride DCl solution (0.01 M), depending on the study. Measurements were taken at time 0 (immediately after placement in the tube) and at 1, 2, and 4 h of incubation. CPMG Inversion-Recovery (IR-CPMG) pulse sequence was applied to obtain 2D T_1_-T_2_ relaxation maps (echo time TE = 60 μs, repetition time RT = 8 s, 38 inversion times evenly distributed on a logarithmic T_1_ scale). Other parameters: dead time DT = 1 μs, number of points = 64,000, pulse length = 12 μs. The FID signal was acquired using the following parameters: DT = 0.5 μs, number of points = 8192, pulse length = 12 μs, RT = 5 s. Relaxometry studies were conducted in a solution of heavy hydrochloric acid DCl at a concentration equivalent to that of fed state gastric fluid (0.01 M).

Data analysis was performed using the Fast Iterative Shrinkage/Thresholding Algorithm (FISTA) algorithm implemented in PROSPA software package, resulting in a 2D T_1_-T_2_ map.

## Figures and Tables

**Table 1:** Qualitative compositions of GRFFOA according to the manufacturer’s declaration. The function of specific substances in the gel raft formulation is presented.

Role of Excipients	Products		
	Formulation A	Formulation B	Formulation C
**Polymer**	Sodium alginate	Sodium alginate	Magnesium alginate
**Viscosity control**	Polyacrylic acid	Carbomer 974P	Xanthan gum
**Surfactant**	-	-	Simethicone
**CO_2_ generating agents**	Sodium bicarbonate Sodium carbonate Calcium carbonate	Sodium bicarbonate Calcium carbonate	Sodium bicarbonate
**Preservatives**	Propylparaben Methylparaben	Propylparaben Methylparaben	Propylparaben Methylparaben
**Flavorings and sweeteners**	Flavoring Sodium saccharin	Mint flavors Sodium saccharin	Natural flavors Honey Fructose
pH control/gelling agent *	-	Sodium hydroxide	Sodium hydroxide
**Colorants**	-	-	Erythrosine (E127)
**Other substances**	-	-	D-panthenol Liquid extracts of *Althaea officinalis * *Papaver rhoeas* Zinc oxide
**Solvent**	Purified water	Purified water	Purified water

* Sodium hydroxide in formulation B neutralizes hydrogen ions on Carbomer chains, enabling their relaxation and gel formation.

**Table 2:** Visual observations of raft behavior in fed state simulated gastric fluid (0.01 M HCl and DCl).

Macroscopic Properties	Products		
	Formulation A	Formulation B	Formulation C
**Coherence of material**	Coherent raft	Coherent raft	No visible raft
**Floating properties**	Rapid raft formation, flotation observed for 2 h (MRI)	Rapid raft formation, flotation observed for 2 h (MRI)	No flotation. Swelling/dissolution of polymer material
**Swelling ability**	Raft swelling after formation	Raft swelling after formation	Polymer swelling without flotation properties

**Table 3:** Relaxometry data summary.

Sample	Time [h]	Peak 1			Peak 2		
		T_1_ [ms]	T_2_ [ms]	T_1_/T_2_	T_1_ [ms]	T_2_ [ms]	T_1_/T_2_
**Formulation A**	0 h	2799	1.88	1489	5301	1274	4.16
	1 h	7430	1.82	4082	5996	2917	2.06
	2 h	88,414	1.87	47,280	8029	2994	2.68
	4 h	-	-	-	5301	1650	3.21
**Formulation B**	0 h	95,541	0.035	2,729,743	3783	867	4.36
	1 h	-	-	-	2475	1665	1.49
	2 h	-	-	-	2408	938	2.57
	4 h	21	0.19	111	3375	1467	2.30
**Formulation C**	0 h	-	-	-	1319	318	4.15
	1 h	-	-	-	3314	760	4.36
	2 h	174	0.04	4350	4397	1410	3.12
	4 h	49	0.19	258	5019	2001	2.51

## Data Availability

The raw data supporting the conclusions of this article will be made available by the authors on request.

## Abbreviations

The following abbreviations are used in this manuscript:

GERD	gastroesophageal reflux disease
GRFFOA	gel raft formulations for oral administration
NMR	nuclear magnetic resonance
MRI	magnetic resonance imaging
MSME	multi-slice multi-echo
CPMG	Carr-Purcell-Meiboom-Gill
IR	inversion-recovery
FID	free induction decay
LF NMR	low-field NMR
